# Economic Burden Associated With Untreated Mental Illness in Indiana

**DOI:** 10.1001/jamahealthforum.2023.3535

**Published:** 2023-10-13

**Authors:** Heather L. Taylor, Nir Menachemi, Amy Gilbert, Jay Chaudhary, Justin Blackburn

**Affiliations:** 1 Department of Health Policy and Management, Indiana University Richard M. Fairbanks School of Public Health, Indianapolis; 2 Regenstrief Institute, Indianapolis, Indiana; 3 Family and Social Services Administration, Indianapolis, Indiana; 4 Wellbeing Informed by Science and Evidence in Indiana, Indianapolis

## Abstract

**Importance:**

There is a paucity of systematically captured data on the costs incurred by society—individuals, families, and communities—from untreated mental illnesses in the US. However, these data are necessary for decision-making on actions and allocation of societal resources and should be considered by policymakers, clinicians, and employers.

**Objective:**

To estimate the economic burden associated with untreated mental illness at the societal level.

**Design, Setting, and Participants:**

This cross-sectional study used multiple data sources to tabulate the annual cost of untreated mental illness among residents (≥5 years old) in Indiana in 2019: the US National Survey on Drug Use and Health, the National Survey of Children’s Health, Indiana government sources, and Indiana Medicaid enrollment and claims data. Data analyses were conducted from January to May 2022.

**Main Outcomes and Measures:**

Direct nonhealth care costs (eg, criminal justice system, homeless shelters), indirect costs (unemployment, workplace productivity losses due to absenteeism and presenteeism, all-cause mortality, suicide, caregiver direct health care, caregiver productivity losses, and missed primary education), and direct health care costs (disease-related health care expenditures).

**Results:**

The study population consisted of 6 179 105 individuals (median [SD] age, 38.0 [0.2] years; 3 132 806 [50.7%] were women) of whom an estimated 429 407 (95% CI, 349 526-528 171) had untreated mental illness in 2019. The economic burden of untreated mental illness in Indiana was estimated to be $4.2 billion annually (range of uncertainty [RoU], $2.1 billion-$7.0 billion). The cost of untreated mental illness included $3.3 billion (RoU, $1.7 billion-$5.4 billion) in indirect costs, $708.5 million (RoU, $335 million-$1.2 billion) in direct health care costs, and $185.4 million (RoU, $29.9 million-$471.5 million) in nonhealth care costs.

**Conclusion and Relevance:**

This cross-sectional study found that untreated mental illness may have significant financial consequences for society. These findings put into perspective the case for action and should be considered by policymakers, clinicians, and employers when allocating societal resources and funding. States can replicate this comprehensive framework as they prioritize key areas for action regarding mental health services and treatments.

## Key Points

**Question:**

How much does untreated mental illness cost society?

**Findings:**

This cross-sectional study of approximately 429 407 Indiana residents found that in 2019, untreated mental illness was associated with $4.2 billion in annual societal costs, consisting of $3.3 billion in indirect costs, $708.5 million in direct health care costs, and $185.4 million in nonhealth care costs.

**Meaning:**

These findings suggest that the financial toll of untreated mental illness at the societal level is substantial and warrants investments in mental health care access, treatments, and workforce.

## Teaser

This cross-sectional study of Indiana residents with untreated mental illness assesses the annual direct and indirect costs to the state’s economy.

## Introduction

In the US, approximately 1 in 5 individuals experiences mental illness each year, yet less than half (46.2%) receive treatment.^1,2^ The increased demand for mental health services has outpaced the provision of care, particularly in the wake of the COVID-19 pandemic,^3^ leaving millions of people untreated or with unmet treatment needs.^4,5^ Untreated and undertreated mental illness is associated with profound long-term consequences, including reduced quality of life, productivity losses, worsening overall health, unemployment, and involvement with the criminal justice system.^1,6^ Furthermore, the inability to receive appropriate treatment for mental illness comes at a great economic burden to individuals, families, and society, and signals missed opportunities or gaps within the health care system.

To prioritize state-level investments and to mobilize action among stakeholders, state policymakers must understand the extent to which untreated mental illness financially affects society, but these data are lacking. Previous cost estimates have assessed the economic burden of specific mental illnesses, such as bipolar disorder,^7^ schizophrenia,^8,9^ and major depressive disorder, but not the costs of untreated illness.^10,11^ Furthermore, other studies focused on costs specifically incurred within health care^12^ have been limited to perinatal mood and anxiety disorders among mothers and children^13^ or were conducted outside the US.^14,15^

Although these previous analyses^7,8,9,10,11,12,13,14,15^ provide an important conceptual framework, the existing body of work fails to systematically capture all indirect costs, such as reduced labor supply, incarceration costs, and homeless shelter costs. This failure ultimately provides a narrow view of the total economic burden, which makes action at the state level more challenging. Comprehensive information that spans mental illnesses, cost-incurring sectors, and populations is needed so that health insurers, policymakers, and employers can gain a full understanding of the economic burden of untreated mental illness. Understanding the more complete economic burden of untreated mental illness puts into context potentially preventable costs that could be averted through improved mental health care access and delivery. This highly relevant information is necessary to inform decisions and galvanize action among a wide array of community-based key stakeholders.

The purpose of the current study was to estimate the economic burden of untreated mental illness in Indiana using a comprehensive approach that can be replicated in other states to inform policy and programs aimed at addressing the burden of untreated mental illness. We extend knowledge on this matter by (1) estimating the individual, family, workplace, and community costs associated with untreated mental illnesses; (2) assessing mental illness broadly (not diagnosis specific); and (3) presenting a societal-level framework to estimate direct and indirect costs that can be replicated in other states. As states look to address issues regarding mental health, including interventions to improve access, reduce disparities, and ensure equity, our approach sought to identify where efforts should be targeted to prevent the greatest economic losses.

## Methods

Because this cross-sectional study used only deidentified and publicly available data, it was deemed exempt by the Indiana University Institutional Review Board (No. 14204) and informed consent was waived. The study adhered to the Strengthening the Reporting of Observational Studies in Epidemiology (STROBE) reporting guideline for cross-sectional studies.

We developed a framework to estimate the annual societal costs associated with untreated mental illness within Indiana using a prevalence-based approach. The overarching premises that underpin this framework are listed in the Box. Essentially, across all outcomes, we assumed that the cost of untreated mental illness was a function of the prevalence of untreated mental illness, the excess number of individuals at risk for experiencing each outcome owing to untreated mental illness, and the costs associated with each outcome (prevalence × risk × costs).

Box. **Underlying Premises Applied to Estimating Costs Associated With Untreated Mental Illness**

- Cost of untreated mental illness is a function of the prevalence of untreated mental illness, the excess people at risk for experiencing each outcome due to untreated mental illness, and the costs associated with each outcome (prevalence × risk × costs).
- Undertreated mental illness is effectively the same as untreated mental illness.
- Treatment of mental illness will alleviate the excess risk of outcomes attributable to untreated mental illness.
- Baseline risk of each outcome attributable to mental illness is proportional to the prevalence of mental illness in the population.
- Excess risk of each outcome attributable to untreated mental illness is calculated using the baseline risk minus published risk estimates.
- Indirect cost estimations:
  - Individuals with mental illness would otherwise have mean wages if not for their illness.
- Direct health care cost estimations:
  - Individuals in Medicaid claims data with a diagnosis code for mental illness, mental health procedure code, or presence of pharmaceutical medications commonly prescribed to treat mental illness.
  - During the year before the first diagnosis, mental health procedure code, or prescribed pharmaceutical medication in Medicaid claims data, individuals experienced sequelae related to their mental illness.
  - For the year before diagnosis/treatment, the individual’s excess health care costs are attributable to their mental illness being untreated.
- Total costs tabulated using Medicaid claims data were multiplied by a conversion factor of 1.7 (95% CI, 1.1-2.1) to estimate comparable direct health care costs for those with private insurance.
- Input parameter assumptions for calculations are available in eMethods 4 and eTable 3 in Supplement 1.

To account for differences in the prevalence and variation in risks of certain outcomes associated with serious mental illnesses (SMI) vs all other mental illnesses (oMI), and mental illnesses among children, we estimated costs separately for each before summing all costs together (eMethods 1 and eTables 1 and 2 in Supplement 1 provide additional details). We identified individual, family, and community outcomes shown to be associated with SMI or oMI in previously published literature of economic analyses of specific mental illnesses.^9,10,13^ These outcomes included (1) direct nonhealth care costs (costs incurred by the criminal justice system and homeless shelters); (2) indirect costs (costs incurred by unemployment, workplace productivity losses through absenteeism [missed days at work] and presenteeism [reduced productivity while at work], all-cause mortality, suicide, caregivers’ direct health care, caregivers’ productivity losses, and missed primary education); and (3) direct health care costs (disease-related health care expenditures). We used 2 different approaches before summing total costs: 1 for estimating direct nonhealth care and indirect costs and 1 for estimating direct health care costs. The full methodologic approach for direct nonhealth care and indirect costs can be found in eMethods 2 in Supplement 1, along with example calculations (eMethods 3 in Supplement 1), and parameter details for these estimations (eMethods 4 and eTable 3 in Supplement 1). The full methodologic approach for direct health care costs can be found in eMethods 5 in Supplement 1. Both approaches required state prevalence data on mental illness and treatment of mental illness described further by the information that follows.

### Prevalence Data

For adults (age ≥18 years), estimates of the prevalence of any mental illness, SMI, treatment of any mental illness, and treatment of SMI were derived from the 2018 to 2019 National Survey on Drug Use and Health (NSDUH).^4^ The NSDUH uses the Kessler K6 scale^16^ to determine nonspecific psychological distress, measuring SMI and any mental illness among respondents. Participants of the NSDUH are also asked if they received treatment or counseling at a mental health clinic or center within the past 12 months.^16^ For children (5-17 years), estimates were derived from the 2018 to 2019 National Survey of Children’s Health (NSCH).^17^ The NSCH is a national interview survey in which parents are asked to report whether their child has any diagnosed emotional, developmental, or behavioral problem, and whether their child received treatment or counseling from a mental health professional.^17^ Both the NSDH and the NSCH use multistage area probability samples to generate national and state representative estimates.

The prevalence of any mental illness and SMI among adults in Indiana based on 2019 NSDUH estimates was 22.6% and 5.9%, respectively.^4^ Furthermore, 26.8% of those with any mental illness and 52.5% of those with SMI responded that they did not receive any mental health treatment within the previous 12 months in Indiana.^4^ To estimate the prevalence of oMI, we subtracted the population with SMI from the total population in Indiana reporting to have any mental illness. Similarly, to determine the number of adults with untreated oMI, we subtracted the number of adults with untreated SMI from the total population of adults who had any untreated mental illness. Based on the 2018 to 2019 NSCH survey, 25.9% of children have a mental illness, of which 4.1% did not receive the treatment that their parent felt they needed in the previous year.^18^

### Statistical Analysis

The number of individuals with untreated mental illness was estimated based on the population in Indiana in 2019^19^ and prevalence estimates of mental illness recorded by the NSDUH and NSCH. All annual excess costs attributable to untreated mental illness among adults and children were summed and adjusted for inflation using the 2019 Consumer Price Index. The overall economic burden of untreated mental illness among adults and children was tabulated as the sum of all direct nonhealth care, indirect, and direct health care costs.

Given the population-level variability in data and lack of 95% CIs available for all point estimates extracted from published literature and government sources, we used an extreme scenario analysis to conduct our sensitivity analyses and manage uncertainty.^20^ In an extreme scenario analysis, each variable is simultaneously set to “take the most optimistic (or pessimistic) value in order to generate a best (or worst) case scenario.”^20^ Thus, to calculate a range of uncertainty (RoU) for each outcome using this methodology, we extracted the lower and upper confidence interval bounds (where possible) provided for all prevalence (Prev), cost (Costs), and excess risk (Excess Risk) estimates used as input parameters for the current study. If a confidence interval was not provided for the point estimate of interest, we extracted the lower and upper range of estimates provided by the source data. The lower bound (LB) of uncertainty for each total cost estimate is the product of the LB prevalence of untreated mental illness, the LB of cost incurred, and the LB number of individuals at excess risk of experiencing the outcome in question: Prev_LB_ × Costs_LB_ × Excess Risk_LB_. Similarly, the upper bound (UB) of uncertainty for each cost estimate is the product of the UB of the prevalence of untreated mental illness, the UB of associated costs incurred, and the UB number of individuals at excess risk for each outcome in question (Prev_UB_ × Costs_UB_ × Excess Risk_UB_). Given the inability to generate 95% CIs for all outcomes extracted from government and published sources, the RoU conservatively estimates the uncertainty associated with the overall results.^20^

The costs for each defined outcome are presented as the excess costs attributable to untreated SMI or oMI. Thus, the total cost estimate was based on the annual sum of costs that Indiana incurs for the average individual with untreated mental illness, adjusted for inflation using the 2019 Consumer Price Index.

Statistical tests were 2-tailed and *P* values < .05 were considered statistically significant. Data analyses were performed from January to May 2022 using SAS, version 9.3 (SAS Institute) and Microsoft Excel were used to tabulate cost estimates.

## Results

The study population consisted of 6 179 105 individuals with a median (SD) age of 38.0 [0.2] years; 3 046 298 (49.3%) men and 3 132 806 (50.7%) women; 18 537 (0.3%) American Indian/Alaska Native/Pacific Islander, 154 478 (2.5%) Asian, 593 194 (9.6%) Black, 444 895 (7.2%) Hispanic/Latine, and 5 116 298 (82.8%) White individuals, per 2019 American Community Survey data.^19^ Based on the prevalence estimates in the NSDUH and NSCH, the population (adults and children) estimated to have any untreated mental illness in Indiana in 2019 was 429 407 (95% CI, 349 526-528 171). The total economic burden of untreated mental illness for Indiana was estimated to be $4.2 billion (RoU, $2.1 billion-$7.1 billion) annually (Table and Figure). On average, each individual affected by untreated mental illness had an associated $9801 (RoU, $5937-$13 454) of costs.

**Table.** Annual Societal Costs Attributable to Untreated Mental Illness in Indiana, 2019

Societal costs	Serious mental illness	Other mental illness	Children	Total
**Direct nonhealth care costs, $**				
Jail usage	3 291 955	2 116 642	NA	5 408 597
Prison usage	66 209 662	101 766 962	2 036 473	101 033 183
Homeless shelter services	4 530 057	3 757 757	NA	8 287 814
Shelter services for long-term homelessness^a^	904 994	750 707	NA	1 655 701
Subtotal	74 936 667	108 392 069	2 036 473	185 365 210
**Indirect costs, $**				
Primary education loss	NA	NA	760 349	760 349
Unemployment	235 812 941	174 895 233	NA	410 708 174
Absenteeism	32 092 960	101 099 598	NA	133 192 559
Presenteeism	152 393 517	589 747 658	NA	742 141 175
All-cause mortality	563 690 748	403 756 056	27 599 655	967 446 804
Suicide	431 324 038	NA	40 446 696	471 770 734
Caregiver productivity loss	211 921 994	305 905 753	NA	517 827 747
Caregiver direct health care	25 266 858	18 123 555	NA	43 390 413
Subtotal	1 652 503 056	1 593 527 853	68 806 700	3 314 837 610
**Direct health care costs, $**				
Medicaid	56 086 373	59 400 367	26 020 849	141 507 589
Private	225 088 188	299 039 237	42 815 039	566 942 465
Subtotal	281 174 561	358 439 604	68 835 888	708 450 053
**All costs totals, $**	2 008 614 286	2 060 359 526	139 679 061	4 208 652 873

Abbreviation: NA, not applicable.

^a^ Individuals with a disabling condition who were homeless consecutively for >1 year or more than 4 times in the past 3 years, with a cumulative time homeless of ≥1 year.

**Figure.**
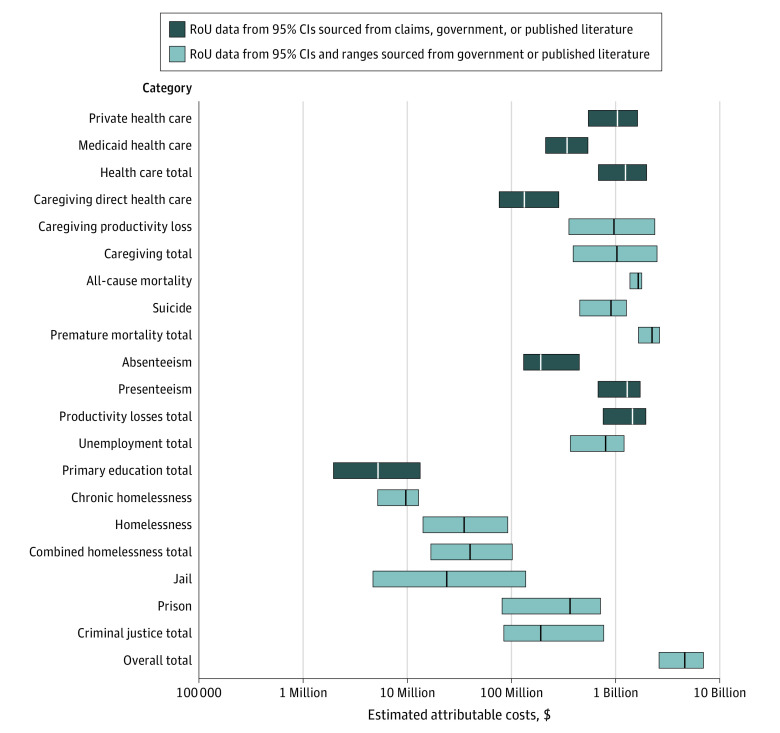
Estimated Costs With Range of Uncertainty (RoU) of Untreated Mental Illness in Indiana, 2019, by Category Error bars represent RoU around each estimated cost point (white or black centerline). Dark blue error bars indicate the RoU from 95% CIs sourced from claims data, government sources, or published literature. Light blue error bars indicate the RoU from 95% CIs and ranges from government sources or published literature.

### Direct Health Care Costs

Excess direct health care costs were estimated to be $708 million annually (RoU, $335 million-$1.2 billion). Of that total, $142 million (RoU, $79 million-$245 million) was paid to Medicaid and $567 million (RoU, $255 million-$990 million) to private insurers.

### Indirect Costs

Based on our estimates, indirect costs attributable to mental illnesses in Indiana totaled $3.3 billion (RoU, $1.7 billion-$5.4 billion). Nearly half of this total cost ($1.5 billion; RoU, 997 million-$1.8 billion) was associated with costs incurred from premature mortality, $995 million of which were derived from the excess risk of unintentional death (RoU, $795 million-$1.1 billion) and $471 million (RoU, $202 million-$719 million) from intentional death or suicide. Other significant indirect costs included productivity losses in the form of absenteeism and presenteeism among those with mental illness ($875 million; RoU, $378 million-$1.2 billion) and productivity losses among those with caregiving responsibilities ($538 million; RoU, $155 million-$1.6 billion).

### Direct Nonhealth Care Costs

Costs incurred through incarceration totaled $175.4 million (RoU, $26.5 million-$441 million). Costs incurred through homeless shelters for services totaled $9.9 million (RoU, $3.4 million-$30.6 million).

## Discussion

We estimated a wide range of costs attributable to untreated mental illness (SMI and oMI), including direct nonhealth care costs, indirect costs, and direct health care costs in Indiana. We found that in 2019, costs were more than $4 billion, making the economic burden of untreated mental illness in Indiana 1.2% of the state’s gross domestic product ($338 billion) in the same year. For context, corn, the leading agricultural commodity for Indiana which accounts for nearly 30% of agricultural production, had $3.8 billion in sales in 2018.^21^ Considering the average wage in Indiana, $4 billion represents approximately 100 000 jobs. Spread across all residents, this is a loss of more than $600 annually for each state resident or nearly $1600 for each family annually.

Overall, these findings help to elucidate the societal burden associated with untreated mental illness. In particular, the lack of sufficient treatment for mental illness was associated with sizeable losses in productive human capital. Given the cost of premature mortality, unemployment, and absenteeism and presenteeism in the workplace, it is evident that employers bear substantial costs associated with untreated mental illness. Thus, there is a business case for employers to support screening for early identification, counseling, and access to and use of mental health services and treatments. These efforts may improve individuals’ productivity and reduce their need for social services.^7,13,22,23^ Costs associated with incarceration and homelessness are also important contributors to the financial burden of untreated mental illness. To reduce the likelihood that individuals with mental illness interact with law enforcement or homeless shelters, experts suggest evidence-based crisis response systems^24^ which have been found to reduce recidivism rates^25^ and to be less costly than standard care.^26^

Individuals who do not receive treatment for their mental illness are also more likely to experience other debilitating physical health conditions.^6^ These physical conditions, which can be exacerbated by untreated mental illness, may be associated with higher overall medical care costs and a greater likelihood of hospitalization.^6,7^ Thus, there is a need for better patient management, early recognition, and continued insurance coverage of mental health treatments.

Clinicians, policymakers, and employers also need to understand the social context and needs associated with mental illness. Given the findings of our study, eliminating barriers to seeking and accessing treatment should be prioritized, including offering culturally responsive care and recognizing populations stigmatized or discriminated against for seeking treatment. Telehealth may be a method to expand access and treatment within Indiana and elsewhere. Although challenges exist with regulatory, licensing, and reimbursement of telehealth and ensuring equitable access, there is increased willingness of clinicians and patients for this delivery modality.^3,24,27^ Furthermore, telehealth outcomes are comparable with in-person care.^3,24,28^ In fact, Indiana recently enabled Medicaid reimbursement for telehealth services to deliver intensive outpatient treatment for psychiatric services.^29^ These measures of intervention may encourage better long-term outcomes and overall cost savings to health care systems and society as whole.^6,30,31^

Other states may benefit from our proposed framework, which may be relatively easy to implement, especially via state−university partnerships.^32^ This proposed framework synthesizes many sources of data into easily interpretable information and translates evidence into a narrative that is more easily understood and preferred by policymakers.^33^ These data can be useful for tracking and evaluating state policies or interventions, including expanding treatment modalities, clinician reimbursements, and other efforts to improve initiation of treatment. Furthermore, having a reproducible framework enables state by state comparisons as well as within states over time. Overall, our framework represents an unbiased approach with methodologic rigor that may increase the reliability and believability of the information for policymakers and other stakeholders.^34^

### Limitations

Although our approach to estimating the state-level burden of untreated mental illness was more comprehensive than most US-based studies, there are relevant limitations to note. First, because there is insufficient evidence to demonstrate that treatments for mental illnesses are fully restorative, it is unlikely that all associated costs described in this study would be greatly reduced or averted with treatment. Nevertheless, evidence-based treatment and interventions to improve access to mental health services should be considered given the potential magnitude of economic costs associated with untreated mental illness. It is also important to note that our approach was not an analysis of the cost-effectiveness of screening and treating mental illness. As such, it is assumed that investments into improving the mental health workforce or service delivery will be required, although the extent of the necessary investments is outside the scope of this study. Second, our framework cannot account for the varying severity levels of mental illness. It may be that those who are diagnosed with mental illness and not receiving treatment may be less ill than those who are diagnosed and are actively receiving treatment. Nevertheless, we calculated a range of uncertainty for all costs, considering varying levels of illness prevalence, costs, and risks. Third, given the high prevalence of SMI and oMI, it is not possible to include all potential negative societal outcomes that incur costs. For example, our study was unable to estimate direct health care costs to Medicare or for judicial and legal service fees associated with incarceration. Nevertheless, through our literature review and use of previously published work on specific mental disorders as guidance,^9,11,12,13^ our approach included the most frequently considered, and presumably the most common, economic societal outcomes associated with mental illness. Fourth, despite efforts to estimate costs across the lifespan, the lack of data for direct health care costs among the Medicare-eligible population implies we underestimated the true economic burden of untreated mental illness when we aggregated costs of the population to the state-level. Fifth, given that the Kessler K6 scale may not capture all individuals who experience mental distress at a subdiagnostic level, but who are nonetheless associated with substantial health and economic burdens,^35^ we may have further underestimated economic burdens. Sixth, the human capital method has its limitations and may contribute to biased estimates because it oversimplifies indirect costs as a value of the mean achievable gross income and does not take into account the nuances of individual social and class status.^36,37^ Seventh, data used in the current study are representative of 2019 and are not reflective of the prevalence of SMI or oMI after the COVID-19 pandemic. Eighth, in cases where Indiana-specific estimates were not available, national estimates were used in our calculations which may not fully represent Indiana’s population. Finally, our analysis was not inclusive of disorders or outcomes associated with substance use in the absence of SMI or oMI, which further suggests that our total cost underestimates the burden of untreated mental illness because we did not factor in behavioral comorbidities.

## Conclusions

This cross-sectional study quantified the economic costs of untreated mental illness. Its findings put into perspective the case for action from a financial perspective and suggest cost savings may be realized by reducing the number of individuals with untreated and undertreated mental illness. These study findings should be considered by policymakers, clinicians, and employers when allocating societal resources and funding. Other states can replicate this comprehensive framework to prioritize key areas for action regarding mental health services and treatments.

## Notes

Supplement 1. **eMethods 1. **Definition of Serious Mental Illness (SMI) and Other Mental Illnesses (oMI) **eTable 1. **International Classification of Disease (ICD-10-CM) Diagnoses Codes for Serious Mental Illness and Serious Emotional Disturbance Used by the State of Indiana **eTable 2. **Categories of International Classification of Disease (ICD-10-CM) Diagnoses Representing Mental Illnesses Included In Analyses **eMethods 2. **Methodological Approach for Estimating Direct Non-Healthcare and Indirect Costs **eMethods 3.** Example Calculations to Estimate Jail Incarceration Costs Attributable to Untreated Serious Mental Illness **eMethods 4. **Details on Direct Non-Healthcare Cost Estimations and Indirect Cost Estimations **eTable 3. **Parameter and Assumption Details on Direct Nonhealthcare Cost Estimations and Indirect Cost Estimations **eMethods 5. **Methodological Approach for Estimating Direct Healthcare Costs
eReferences.

Supplement 2.
Data Sharing Statement
