# Supplementary material for: Economic Burden Associated With Untreated Mental Illness in Indiana
Source: JAMA Health Forum. 2023 Oct 13;4(10):e233535. doi: 10.1001/jamahealthforum.2023.3535 (PMC10576212; doi:10.1001/jamahealthforum.2023.3535)
Supplement: Supplement 1. — eMethods 1. Definition of Serious Mental Illness (SMI) and Other Mental Illnesses (oMI) eTable 1. International Classification of Disease (ICD-10-CM) Diagnoses Codes for Serious Mental Illness and Serious Emotional Disturbance Used by the State of Indiana eTable 2. Categories of International Classification of Disease (ICD-10-CM) Diagnoses Representing Mental Illnesses Included In Analyses eMethods 2. Methodological Approach for Estimating Direct Non-Healthcare and Indirect Costs eMethods 3. Example Calculations to Estimate Jail Incarceration Costs Attributable to Untreated Serious Mental Illness eMethods 4. Details on Direct Non-Healthcare Cost Estimations and Indirect Cost Estimations eTable 3. Parameter and Assumption Details on Direct Nonhealthcare Cost Estimations and Indirect Cost Estimations eMethods 5. Methodological Approach for Estimating Direct Healthcare Costs eReferences. [file jamahealthforum-e233535-s001.pdf]

Supplemental Online Content

Taylor HL, Menachemi N, Gilbert A, Chaudhary J, Blackburn J. Economic burden associated with untreated mental illness in Indiana. *JAMA Health Forum*. 2023;4(3):e233535. doi:10.1001/jamahealthforum.2023.3535

eMethods 1. Definition of Serious Mental Illness (SMI) and Other Mental Illnesses (oMI)

eTable 1. International Classification of Disease (ICD-10-CM) Diagnoses Codes for Serious Mental Illness and Serious Emotional Disturbance Used by the State of Indiana

eTable 2. Categories of International Classification of Disease (ICD-10-CM) Diagnoses Representing Mental Illnesses Included In Analyses

eMethods 2. Methodological Approach for Estimating Direct Non-Healthcare and Indirect Costs

eMethods 3. Example Calculations to Estimate Jail Incarceration Costs Attributable to Untreated Serious Mental Illness

eMethods 4. Details on Direct Non-Healthcare Cost Estimations and Indirect Cost Estimations

eTable 3. Parameter and Assumption Details on Direct Nonhealthcare Cost Estimations and Indirect Cost Estimations

eMethods 5. Methodological Approach for Estimating Direct Healthcare Costs

eReferences.

This supplemental material has been provided by the authors to give readers additional information about their work.

© 2023 Taylor HL et al. *JAMA Health Forum.*

eMethods 1: Definition of Serious Mental Illness (SMI) and other mental illnesses (oMI)

There are multiple existing definitions of mental illnesses, including SMI.[1] For instance, the National Institute of Mental Health defines SMI as "any mental, behavioral, or emotional disorder resulting in serious functional impairment."[2] The National Committee on Quality Assurance (NCQA) defines individuals with SMI as those who meet at least one of the following criteria within the measurement period: (1) at least one acute inpatient claim/encounter with any diagnosis of schizophrenia, bipolar I disorder, or major depression, OR; (2) at least two visits in an outpatient, intensive outpatient (IOP), partial hospitalization (PH), emergency department (ED), or nonacute inpatient setting, on different dates of service, any diagnosis of schizophrenia, OR; (3) at least two visits in an outpatient, intensive outpatient, partial hospitalization, emergency department, or nonacute inpatient setting on different dates of service with a diagnosis of bipolar I disorder.[3] Although we acknowledge differences in existing definitions of mental illnesses,[1] including SMI, for the purposes of the current analysis we defined SMI conditions consistent with how it is defined by the Indiana FSSA (see eTable 1).

© 2023 Taylor HL et al. *JAMA Health Forum.*

eTable 1. International Classification of Disease (ICD-10-CM) diagnoses codes for serious mental illness and serious emotional disturbance used by the State of Indiana

Serious Mental Illness	Serious Emotional Disturbance
F20.xx (Schizophrenia and sub codes up to 2 places)<br>F25.xx (Schizoaffective Disorder and sub codes up to two places)<br>F31.xx (Bipolar and all sub codes up to 2 places)<br>F33.xx (Major depression Recurrent and all sub codes up to two places)	F20 (Schizophrenia)<br>F31.0 (Bipolar Disorder: Type I & II)<br>F31.1 (Current manic episode without psychotic)<br>F31.2 (Psychosis)<br>F31.9 (Unspecified)<br>F32.9 (Major Depression- Single episode)<br>F33 (Major depression Recurrent)<br>F33 (Major depression Recurrent)<br>F34.81 (Disruptive mood dystregulation disorder)<br>F43.1 (PTSD)<br>F50.0 (Anorexia)<br>F50.2 (Bulimia)<br>F63.9 (Impulse Control Disorder)<br>F84.0 (Autistic disorder)<br>F90 (attention-deficit/hyperactivity disorder, Conduct Disorder, Oppositional Defiant Disorder)

We also referenced the Healthcare Cost and Utilization Project (H-CUP) Clinical Classification Software Refined (CCSR) categories of mental, behavioral and neurodevelopmental disorders to identify associated International Classification of Disease version 10 (ICD-10) codes. The selected CCSR categories were grouped into larger categories representing SMI (such as schizophrenia and bipolar disorder) and other mental disorders consistent with the work of Breslau et al (2021).[4] eTable 2 displays Breslau et al's groupings. The full CCSR reference file with ICD-10 is available from H-CUP (https://www.hcup-us.ahrq.gov/toolssoftware/ccsr/DXCCSR-Reference-File-v2022-1.xlsx).

© 2023 Taylor HL et al. *JAMA Health Forum.*

eTable 2. Categories of International Classification of Disease (ICD-10-CM) diagnoses representing mental illnesses included in analyses

Clinical Classification System Revised Category	Mental Illness Category
Schizophrenia spectrum and other psychotic disorders (CCSR MBD001)	Schizophrenia or Other Psychotic Disorder
Bipolar and related disorders (CCSR MBD003)	Bipolar Disorder
Other specified and unspecified mood disorders (CCSR MBD004)	Depression or Other Mood Disorder
Depressive disorders (CCSR MBD002)	
Anxiety and fear-related disorders (CCSR MBD005)	Anxiety or Stress-Related Disorder
Trauma- and stressor-related disorders (CCSR MBD007)	
Obsessive-compulsive and related disorders (CCSR MBD006)	
Disruptive, impulse-control and conduct disorders (CCSR MBD008)	Other Mental Illness
Personality disorders (CCSR MBD009)	
Feeding and eating disorders (CCSR MBD010)	
Somatic disorders (CCSR MBD011)	
Suicidal ideation/attempt/intentional self-harm (CCSR MBD012)	
Suicide attempt/intentional self-harm; subsequent encounter (CCSR MBD027)	
Miscellaneous mental and behavioral disorders/conditions (CCSR MBD013)	

Adapted from Breslau et al, 2021.[4]

eMethods 2: Methodological approach for estimating direct non-healthcare and indirect costs

Our framework for estimating direct non-healthcare and indirect costs consisted of a series of equations for each outcome we identified. First, because not all individuals with SMI or oMI experience an outcome due to their illness, we had to estimate the excess risk of an individual experiencing the outcome attributable to their SMI or oMI. This was calculated by subtracting the risk of the outcome among the general population from the risk of the outcome among those with SMI or oMI. Next, the excess risk was multiplied by the total population in Indiana with SMI or oMI, the expected untreated or undertreated proportion of the SMI or oMI population,[5,6] and the annual cost of each outcome per person. We assumed that the untreated proportion of those with SMI or oMI in each outcome is proportional to the untreated proportion of SMI or oMI in the general population of Indiana. Each direct non-healthcare and indirect outcome was estimated using the steps described above. An example as to how we walked through each calculation is provided in Supplemental Material eMethods3.

To estimate costs attributable to premature mortality (all-cause mortality and suicide), an additional method was used – the human capital method.[7] The human capital method estimates the economic value of an average individual's future earnings had they not missed work due to death or illness.[7] Thus, we estimated the gross income lost among those that died prematurely before the retirement age of 65. Specifically, we used the Centers for Disease Control and Prevention WONDER dataset[8] to determine the number of individuals in Indiana who died due to any causes (other than intentional harm) before the age of 65 in 2019. For each death, we tabulated the number of working years lost (between the ages of 18 and 64) and multiplied it by the excess number of individuals with SMI or oMI and the average 2019 annual wage in Indiana.

© 2023 Taylor HL et al. *JAMA Health Forum.*

For deaths due to intentional harm or suicide (ICD-10-Clinical Modification Codes X71-X83), we tabulated the total number of working years lost and multiplied this number by the excess number of individuals with SMI or oMI and average 2019 annual wage in Indiana.[9]

Finally, specific details and parameters used for the estimation of each direct non-healthcare and indirect outcome, including premature all-cause mortality and suicide, can be found in Supplemental Material eMethods4, eTable3. Our framework for estimating direct non-healthcare and indirect costs, including model inputs and specific sources, can be found at https://hdl.handle.net/1805/34678.

© 2023 Taylor HL et al. *JAMA Health Forum.*

eMethods 3: Example calculations to estimate jail incarceration costs attributable to untreated serious mental illness

For example, to estimate jail incarceration costs attributable to untreated SMI, we began with the 2019 adult jail population (n=20,880),[10] the proportion of Indiana's jail population with SMI (26%),[11] the population in Indiana with SMI (n=296,000),[5] and the general adult population in Indiana without SMI (n=4,733,000).[5] These data allowed us to determine the risk of jail incarceration among the general Indiana adult population (0.003) and the risk of jail incarceration among adults with SMI (0.018). Then we subtracted the risk of jail incarceration among the general population from the risk of jail incarceration among those with SMI to calculate the excess risk of jail incarceration attributable to SMI (0.015). The excess risk (0.015) was then multiplied by the adult population in Indiana with SMI (n=296,000), the proportion of adults in Indiana with untreated SMI (0.525), the average length of stay per Indiana jail resident (26 days),[12] and the average daily cost of incarceration per jail resident in Indiana ($54.00),[13] to estimate the total costs of jail incarceration attributable to adults with untreated SMI to be $3,291,954.63.

© 2023 Taylor HL et al. *JAMA Health Forum.*

eMethods 4: Details on direct non-healthcare cost estimations and indirect cost estimations

All estimates are based on 2019 Indiana specific parameters derived from the literature and government sources. Excess costs for each outcome listed below were calculated by multiplying the excess number of individuals with serious mental illness (SMI) or other mental illness (oMI) who experience the outcome, the proportion of this population assumed to be undertreated or untreated,[5,6] and the cost of each outcome per person within a 12-month timeframe. Details and sources related to each outcome estimation are described below and listed in eTable 3.

Direct non-healthcare costs

Criminal Justice System – Costs incurred within jails, prisons, and juvenile detention centers were estimated based on the general population incarcerated,[10,14] the excess number of individuals with SMI and oMI in each of these correctional facilities,[11,15,16] the average length of stay per correctional facility,[12,17,18] and the annual cost per day per resident within each correctional facility.[13,19,20] The excess criminal justice system costs attributable to untreated mental illness were calculated by multiplying the annual cost of incarceration, the excess number of individuals incarcerated with SMI and MI, and the expected proportion who are untreated based on NSDUH or NSCH data.[5,6]

Homeless services – Costs associated with homeless shelters were estimated based on the general homeless population,[21] the prevalence of SMI and oMI residing in homeless shelters,[22,23] the average length of stay in a homeless shelter,[24] and the costs per day per individual in a homeless shelter.[25,26] Costs were separately calculated for the proportion of the population who are chronically homeless (have at least one disability and on average are homeless for at least one year).[23] The excess homeless service costs attributable to untreated mental illness were calculated by multiplying the annual cost of

an individual residing in a homeless shelter, the excess number of homeless individuals with SMI and MI, and the expected proportion who are untreated based on NSDUH or NSCH data.[5,6]

Indirect Costs

Unemployment – Costs associated with unemployment were estimated based on the total labor force in Indiana,[27] the proportion unemployed in Indiana (ages 18-64),[27] the excess number of individuals unemployed with SMI or oMI,[28] and the average mean wage in Indiana.[9] The excess unemployment costs attributable to untreated mental illness were calculated by multiplying the annual lost wages due to unemployment, the excess number of individuals unemployed with SMI and MI, and the expected proportion who are untreated based on NSDUH.[5]

Workplace productivity losses –Costs for absenteeism were based on the number of individuals with full-time employment and SMI or MI,[27,29] the mean hourly wage for Indiana,[9] and the average excess total hours absent per year attributable to SMI & MI conditions.[30,31] Estimates for presenteeism were based on the number of individuals with full-time employment and SMI or MI,[27,29] the mean hourly wage for Indiana,[9] and the average excess total hours of lost productivity in a year attributable to SMI & MI conditions.[30,31] The excess workplace productivity losses attributable to untreated mental illness were calculated by multiplying the annual cost of excess hours of absenteeism and presenteeism per individual employed with SMI or MI and the expected proportion who are untreated based on NSDUH.[5]

Premature mortality – All-cause mortality – Costs associated with all-cause mortality were estimated using the human capital method among individuals who died before the

© 2023 Taylor HL et al. *JAMA Health Forum.*

age of 65 for any cause other than intentional harm. All-cause mortality in Indiana was obtained from the Centers for Disease Control and Prevention WONDER dataset for the year of 2019. Costs associated with all-cause mortality were based on the average mortality rate for the general population,[32] the risk of premature death among those with SMI or oMI,[33] and the average wage of working adults in Indiana.[9] We calculated the total years lost to premature mortality from age 18 to 65 for each individual identified. Excess costs associated with all-cause premature mortality attributable to untreated mental illness were calculated by multiplying the total years of life lost among the excess number of individuals with SMI or MI who died prematurely and the average annual wage in Indiana.

Premature mortality – Suicide – Costs associated with suicide were estimated using the human capital method among individuals (ages 5-64) whose underlying cause of death in 2019 was suicide (deaths from intentional harm). This information was obtained from the Centers for Disease Control and Prevention WONDER dataset.[34] We attributed all suicide deaths to untreated mental illness and estimated the total years lost to suicide from age 18 to 65 for each individual identified. Total years lost for the entire population was then multiplied by the average annual wage in Indiana.[9]

Caregivers' productivity loss – Cost estimates were based the number of caregivers in Indiana,[35] the proportion of individuals with SMI or oMI who require a caregiver,[36,37] the mean hourly wage,[9] and the annual excess hours caregivers spend devoting time to family with an SMI or oMI.[36] The excess caregiver productivity losses attributable to untreated mental illness were calculated by multiplying the number of individuals with SMI and MI

© 2023 Taylor HL et al. *JAMA Health Forum.*

who require family caregiving, the annual cost of excess hours lost due to caregiving, and the expected proportion who are untreated based on NSDUH.[5]

Caregivers' excess direct healthcare costs - Cost estimates were based the number of caregivers in Indiana,[35] the proportion of individuals with SMI or oMI who require a caregiver,[36,37] the mean hourly wage,[9] and the average excess healthcare costs of caregivers who spend devoting time to family with an SMI or oMI.[38-40] The excess caregiver productivity losses attributable to untreated mental illness were calculated by multiplying the number of individuals with SMI and MI who require family caregiving, the caregivers' average annual excess healthcare costs, and the expected proportion who are untreated based on NSDUH.[5]

Primary education funding losses - The excess primary education funding losses attributable to untreated mental illness were calculated by multiplying the number of school-age children who experience any MI,[6] excess number of days children with SMI and MI are absent from school due to their illness,[41] Indiana's funding contribution to primary education per student per day,[42] and the expected proportion who are untreated based on NSCH.[6]

© 2023 Taylor HL et al. *JAMA Health Forum.*

eTable 3. Parameter and Assumption Details on direct non-healthcare cost estimations and indirect cost estimations

Parameter	Details	Type	Source
Mental illness			
5.9% (95% CI: 4.5% to 7.7%)	SMI[a] Indiana adults	Prevalence	NSDUH
3.1% (95% CI: 2.4% to 4.0%)	Untreated SMI[a] Indiana adults	Prevalence	NSDUH
22.7% (95% CI: 20.1% to 25.6%)	oMI[b] Indiana adults	Prevalence	NSDUH
6.1% (95% CI: 5.4% to 6.9%)	Untreated oMI[b] Indiana adults	Prevalence	NSDUH
25.9% (95% CI: 22.6% to 29.5%)	MI[c] Indiana children	Prevalence	NSCH
4.1% (95% CI: 2.3% to 7.1%)	Untreated MI[c] Indiana children	Prevalence	NSCH
Criminal Justice System (Direct non-healthcare costs)			
4,462 adults (95% CI: 4,329 to 4,733)	Excess number of adults in jail with SMI[a]	Risk	Government report
5,625 adults[d]	Excess number of adults in jail with oMI[b]	Risk	Literature
$54.00 (Range: $22.00 to $276.00)	Per diem for jail resident in Indiana	Cost	Literature
26 days (Range: 10.1 to 35.9 days)	Average length of stay in jail	Cost	Government report
2,430 (95% CI: 2,171 to 2,689)	Excess number of individuals in prison with SMI[a]	Risk	Government report
7,323 adults[d]	Excess number of individuals in prison with oMI[b]	Risk	Government report
$52.62[d]	Per diem prison resident in Indiana	Cost	Government website
2.7 years (Range: 6m to 5 years)	Average length of stay in Indiana prison	Cost	Government report
231 children (Range: 194 to 241)	Excess number of children in juvenile detention with MI[c]	Risk	Literature
$232.13[d]	Per diem for juvenile detention resident in Indiana	Cost	Government report
240 days[d]	Average length of stay in Indiana juvenile detention center	Cost	Report
Homelessness (Direct non-healthcare costs)			
1,061 individuals (95% CI: 783 to 1360)	Excess number of individuals experiencing homelessness in Indiana with SMI[a]	Risk	Report
1,725 individuals[d]	Excess number of individuals experiencing homelessness in Indiana with oMI[b]	Risk	Literature
141 days (Range: 63 to 339 days)	Median number of days a person is homeless	Cost	Literature

© 2023 Taylor HL et al. *JAMA Health Forum.*

$57.66.[d]	Daily cost of homelessness/person in Indiana	Cost	Report
82 individuals (95% CI: 60 to 105)	Excess number of individuals experiencing chronic homelessness in Indiana with SMI[a]	Risk	Government website
133 individuals[d]	Excess number of individuals experiencing chronic homelessness in Indiana with oMI[b]	Risk	Literature
365 (Range: 8m to 1yr)	Average number of days person is chronically homeless	Cost	Literature
Primary Education (Indirect costs)			
7.7 days (95% CI: 6.3 to 9.1)	Excess days absent due to MI[c]	Risk	Literature
$51.08[d]	Indiana cost per student per day	Cost	Government website
Unemployment (Indirect costs)			
9,596 adults (95% CI: 9,029 to 10,022)	Excess number of adults experiencing unemployment in Indiana with SMI[a]	Risk	Literature
13,953 adults (10,358 to 16,689)	Excess number of adults experiencing unemployment in Indiana with oMI[b]	Risk	Literature
$46,770 (95% CI: $25,670 to $56,250)	Average wage of an employee in Indiana	Cost	Government website
Absenteeism (Indirect Costs)			
$22.49 (95% CI: $12.32 to $27.05)	Mean hourly wage in Indiana	Cost	Government website
66.8 hours (95% CI: 20.8 to 62.4)	Excess hours per year lost due to SMI[a] absenteeism	Risk	Literature
31.2 hours (95% CI: 20.8 to 41.6)	Excess hours per year lost due to oMI[b] absenteeism	Risk	Literature
Presenteeism (Indirect Costs)			
$22.49 (95% CI: $12.32 to $27.05)	Mean hourly wage in Indiana	Cost	Government website
317 hours (95% CI: 187 to 452)	Excess hours per year lost due to SMI[a] presenteeism	Risk	Literature
182 hours (95% CI: 156 to 208)	Excess hours per year lost due to oMI[b] presenteeism	Risk	Literature
Premature mortality (Indirect Costs)			
1,752 adults (95% CI: 729 to 3,363)	Excess deaths attributable to SMI[a]	Risk	CDC Wonder; Literature

© 2023 Taylor HL et al. *JAMA Health Forum.*

2,470 adults (95% CI: 1,801 to 3,125)	Excess risk of dying prematurely due to oMI[b] among adults	Risk	CDC Wonder; Literature
79 children[d]	Excess risk of dying prematurely due to MI[c] among children	Risk	CDC Wonder; Literature
$46,770 (95% CI: $25,670 to $56,250)	Average wage of an employee in Indiana	Cost	Government website
Suicide (Indirect Costs)			
17,552 (95% CI: 11,764 to 32,062)	Excess number of years lost due to suicide attributable to MI[c]	Risk	CDC Wonder
$46,770 (95% CI: $25,670 to $56,250)	Average wage of an employee in Indiana	Cost	Government website
Caregiving Productivity Losses (Indirect Costs)			
385 hours (Range: 239 to 811)	Excess number of hours spent caring for individuals with MI[c]	Risk	Literature
$22.49 (95% CI: $12.32 to $27.05)	Mean hourly wage in Indiana	Cost	Government website
Caregivers Excess Healthcare Costs (Indirect Costs)			
$1,031 ($471.69 to $2,727.58)	Excess healthcare costs for individuals caring for SMI[a] caregivers	Risk and Costs	Literature
$512.72 ($428.48 to $595.92)	Excess healthcare costs for oMI[b] caregivers	Risk and Costs	Literature

Source: Authors analysis of annual societal costs attributable to untreated mental illness, as described in the text.

[a] – Serious mental illness

[b] – other mental illness (excluding serious mental illness)

[c] – any mental illness

[d] – point estimate with no confidence interval or range data available

© 2023 Taylor HL et al. *JAMA Health Forum.*

eMethods 5: Methodological approach for estimating direct healthcare costs

To estimate direct healthcare costs of untreated SMI and oMI, we used a retrospective matched cohort design using Indiana Medicaid claims. First, we created a cohort of enrollees (age 5 years and older) with at least 24 months of continuous enrollment spanning calendar years 2018 and 2019. Next, we identified which of those enrollees had a SMI or oMI using a combination of primary diagnosis codes, mental health-related procedure codes as defined by the Indiana Health Coverage Program Fee Schedule,[43] or the presence of pharmaceutical medications commonly prescribed to treat SMI or oMI according to the National Committee on Quality Assurance HEDIS® 2019 Medication List Directory of National Drug Codes.[44] Next, we identified enrollees who met our definition of SMI or oMI in their second 12 months of enrollment but not during their first 12 months. Thus, if the enrollee was receiving treatment or services in their first year of enrollment, as indicated by claims with primary diagnosis codes, procedure codes, or medications associated with SMI or oMI, they were not included. Next, we matched enrollees (who met our inclusion criteria) by sex, age, race, and county of residence to individuals with no primary diagnosis codes, procedure codes, or medications associated with SMI or oMI during the entire 24-month. Any individual who reached age 64 before December 31, 2019, was also excluded since Medicare incurred costs are not observable in these data.

We assumed that those who received treatment for SMI or oMI experienced sequelae of the illness for some period prior to the diagnosis, procedures, or medication.[45,46] Therefore, the difference in costs post-treatment versus pre-treatment represent costs attributable to untreated or undertreated SMI or oMI. Health service costs accrued in the year prior to SMI or oMI treatment, and costs accrued in the first year of treatment were totaled. Total health care expenditures, including inpatient care, outpatient care, emergency departments, pharmacy costs, and other

© 2023 Taylor HL et al. *JAMA Health Forum.*

healthcare services were tabulated. Since our focus was on all excess costs beyond mental health treatment, any claims with a SMI or oMI procedure, medication, or diagnosis were excluded from the total costs. Among enrollees meeting the definition of having untreated SMI or oMI, we subtracted the total annual healthcare costs in year 2 from year 1, and likewise for the matched comparison group. We then took the difference in costs from the untreated SMI or oMI group and the matched comparison group to estimate the cost of untreated disease. To extrapolate costs to privately insured individuals, we multiplied the cost of unmanaged/untreated disease among the Medicaid population by a conversion factor of 1.7 (95% CI: 1.1 to 2.1), which has been previously used to convert Medicaid costs of MI to private insurance.[47,48] Finally, to capture the total excess direct healthcare costs attributable to untreated and undertreated SMI or oMI, we multiplied the excess cost of SMI or oMI by the proportion of the statewide population expected to have untreated SMI or oMI.

© 2023 Taylor HL et al. *JAMA Health Forum.*

eReferences

1. Buck JA. Problems With 'Serious Mental Illness' As A Policy Construct. *Health Aff*. 2021;40(12):1953-1960.
2. Mental Illness. National Institute of Mental Health (NIMH). https://www.nimh.nih.gov/health/statistics/mental-illness. Accessed June 13, 2022
3. Monitoring Metrics for Section 1115 Demonstrations with SMI/SED Policies. Medicaid.gov https://www.medicaid.gov/medicaid/section-1115-demo/downloads/evaluation-reports/smi-monitoring-metrics.pdf.
4. Breslau J, Dana B, Pincus H, Horvitz-Lennon M, Matthews L. Empirically identified networks of healthcare providers for adults with mental illness. *BMC Health Serv Res*. 2021;21(1):777.
5. 2019 National Survey of Drug Use and Health (NSDUH) Releases. Substance Abuse and Mental Health Services Administration https://www.samhsa.gov/data/release/2019-national-survey-drug-use-and-health-nsduh-releases. Accessed June 13, 2022.
6. National Survey of Children's Health 2018-2019: Children with mental, emotional, developmental or behavioral problems, Indiana. https://www.childhealthdata.org/browse/survey/results?q=7981&r=16. Accessed June 27, 2022.
7. Kirch W, ed. Human Capital Approach. In: Encyclopedia of Public Health. Dordrecht: Springer Netherlands; 2008:697-698.
8. Multiple Cause of Death Data on CDC WONDER. Centers for Disease Control and Prevention. https://wonder.cdc.gov/mcd.html. Accessed January 1, 2022.

© 2023 Taylor HL et al. *JAMA Health Forum.*

9. Occupational Employment and Wage Estimates - May 2019. U.S. Bureau of Labor Statistics. https://www.bls.gov/oes/2019/may/oes_in.htm. Published September 9, 2008. Accessed January 2, 2022.
10. Indiana 2019. National Institute of Corrections. https://nicic.gov/state-statistics/2019/indiana-2019. Published May 25, 2021. Accessed July 7, 2022.
11. Bronson J, Berzofsky BSM, H. P. Indicators of Mental Health Problems Reported by Prisoners and Jail Inmates, 2011-12. https://bjs.ojp.gov/content/pub/pdf/imhprpji1112.pdf.
12. Zeng Z, Minton TD, Statisticians B. Jail Inmates in 2019. https://bjs.ojp.gov/content/pub/pdf/ji19.pdf. Published 2021. Accessed June 29, 2022.
13. What Jails Cost - Indiana. Vera Institute. https://www.vera.org/publications/what-jails-cost-statewide/indiana. Accessed January 2, 2022.
14. Indiana Department of Correction. Wikipedia, The Free Encyclopedia. https://en.wikipedia.org/w/index.php?title=Indiana_Department_of_Correction&oldid=1091382852. Published June 3, 2022.
15. *Mental Health in Indiana. National Alliance on Mental Illness; 2021. https://www.nami.org/NAMI/media/NAMI-Media/StateFactSheets/IndianaStateFactSheet.pdf.*
16. Intersection between mental health and the Juvenile Justice System. Office of Juvenile Justice and Deliquency Prevention. https://ojjdp.ojp.gov/model-programs-guide/literature-reviews/intsection_between_mental_health_and_the_juvenile_justice_system.pdf. Accessed August 2, 2022.

© 2023 Taylor HL et al. *JAMA Health Forum.*

17. Pearman FA, Curran FC, Fisher B, Gardella J. Are Achievement Gaps Related to Discipline Gaps? Evidence From National Data. *AERA Open*. 2019;5(4):2332858419875440.

18. Kaeble D, Statistician B. Time served in state prison, 2018. https://bjs.ojp.gov/content/pub/pdf/tssp18.pdf. Published 2021. Accessed January 2, 2022.

19. How much does it cost to keep an offender in prison? https://faqs.in.gov/hc/en-us/articles/115005238288-How-much-does-it-cost-to-keep-an-offender-in-prison-. Accessed January 2, 2022.

20. Fiscal Year 2019 Juvenile Facilities Per Diem Report. Indiana Deparment of Correction. https://www.in.gov/idoc/files/FY19-Per-Diem.pdf. Accessed April 3, 2022.

21. Homeless in Indiana Statistics 2019. Homeless Estimation by State. https://www.usich.gov/homelessness-statistics/in/. Accessed January 3, 2022.

22. Homelessness and Mental Illness: A Challenge to Our Society. Brain & Behavior Research Foundation. https://www.bbrfoundation.org/blog/homelessness-and-mental-illness-challenge-our-society. Published November 19, 2018. Accessed April 13, 2022.

23. Homelessness in Indianapolis. Indiana University Public Policy Institute, Center for Research on Inclusion and Social Policy. https://policyinstitute.iu.edu/doc/pit-count-2021.pdf. Accessed August 2, 2022.

24. Cobb-Clark DA, Herault N, Scutella R, Tseng YP. A journey home: What drives how long people are homeless? *J Urban Econ*. 2016;91:57-72.

© 2023 Taylor HL et al. *JAMA Health Forum.*

25. Laura Littlepage AR. *Estimated Costs of Homelessness in Indianapolis in 2016*. Indiana University Public Policy Institute; 2018. https://ppidb.iu.edu/Uploads/PublicationFiles/cost-of-homelessness-2016.pdf.
26. Mental illness and homelessness. National Coalition for the Homeless https://nationalhomeless.org/factsheets/Mental_Illness.pdf. Accessed January 7, 2022.
27. July Indiana Employment Report. Indiana Department of Workforce Development. https://content.govdelivery.com/accounts/INDWD/bulletins/258882e. Accessed July 7, 2022.
28. Projections of National Expenditures for Treatment of Mental and Substance Use Disorders, 2010-2020. https://store.samhsa.gov/product/Projections-of-National-Expenditures-for-Treatment-of-Mental-and-Substance-Use-Disorders-2010-2020/SMA14-4883. Accessed August 2, 2022.
29. Luciano A, Meara E. Employment status of people with mental illness: national survey data from 2009 and 2010. *Psychiatr Serv*. 2014;65(10):1201-1209.
30. Gardner HH, Kleinman NL, Brook RA, Rajagopalan K, Brizee TJ, Smeeding JE. The economic impact of bipolar disorder in an employed population from an employer perspective. *J Clin Psychiatry*. 2006;67(8):1209-1218.
31. Stewart WF, Ricci JA, Chee E, Hahn SR, Morganstein D. Cost of lost productive work time among US workers with depression. *JAMA*. 2003;289(23):3135-3144.
32. Indiana. America’s Health Rankings. https://www.americashealthrankings.org/learn/reports/2019-annual-report/state-summaries-indiana. Accessed July 8, 2022.

© 2023 Taylor HL et al. *JAMA Health Forum.*

33. de Mooij LD, Kikkert M, Theunissen J, et al. Dying Too Soon: Excess Mortality in Severe Mental Illness. *Front Psychiatry*. 2019;10:855.
34. Multiple Cause of Death Data on CDC WONDER. Centers for Disease Control and Prevention. https://wonder.cdc.gov/mcd.html. Accessed January 1, 2022.It's National Family Caregivers Month. Indiana Ranks Last in the Nation. http://www.iaaaa.org/its-national-family-caregivers-month-indiana-ranks-last-in-the-nation/. Accessed July 8, 2022.
35. It's National Family Caregivers Month. Indiana Ranks Last in the Nation. Indiana Association of Area Agencies on Aging. http://www.iaaaa.org/its-national-family-caregivers-month-indiana-ranks-last-in-the-nation/. Accessed July 8, 2022.
36. On Pins and Needles: Caregivers of Adults with Mental Illness. National Alliance for Caregiving; 2016. https://www.caregiving.org/wp-content/uploads/2020/05/NAC_Mental_Illness_Study_2016_FINAL_WEB.pdf.
37. Corson T. The Impact of Perceived Mental Illness Stigma on Caregivers' Desire to Relinquish Care. 2017. https://scholarscompass.vcu.edu/cgi/viewcontent.cgi?article=5791&context=etd.
38. Chatterton, Mary Lou, Xiongkan Ke, Barbara Edelman Lewis, Krithika Rajagopalan, and Arthur Lazarus. 2008. "Impact of Bipolar Disorder on the Family: Utilization and Cost of Health Care Resources." P & T: A Peer-Reviewed Journal for Formulary Management 33 (1): 15–34.
39. Kuvadia, Harsh, Ian A. Beren, H. Lynn Starr, John J. Sheehan, Nathan L. Kleinman, and Richard A. Brook. 2021. "Direct and Indirect Costs Among Caregivers of Patients With

© 2023 Taylor HL et al. *JAMA Health Forum.*

Major Depressive Disorder and Suicidal Ideation or Suicidal Attempt.” The Primary Care Companion to CNS Disorders 23 (4). https://doi.org/10.4088/PCC.20m02893.

40. Ray, G. Thomas, Constance M. Weisner, Cosette J. Taillac, and Cynthia I. Campbell. 2017. “The High Price of Depression: Family Members’ Health Conditions and Health Care Costs.” General Hospital Psychiatry 46 (May): 79–87..
41. Lawrence D, Dawson V, Houghton S, Goodsell B, Sawyer MG. Impact of mental disorders on attendance at school. *Aust J Edu Chem*. 2019;63(1):5-21.
42. Indiana. Indiana Department of Education. https://inview.doe.in.gov/state/1088000000/finance. Accessed July 8, 2022.
43. Indiana Health Coverage Programs (IHCP) Fee Schedules. Indiana Medicaid. http://provider.indianamedicaid.com/ihcp/Publications/MaxFee/fee_home.asp. Accessed July 26, 2022.
44. Funk L. HEDIS 2019 Medication List Directory (MLD) of NDC codes. https://www.ncqa.org/hedis/measures/hedis-2019-ndc-license/. Accessed July 7, 2022.
45. Panzarino PJ Jr. The costs of depression: direct and indirect; treatment versus nontreatment. *J Clin Psychiatry*. 1998;59 Suppl 20:11-14.
46. Behavioral Health: Research on Health Care Costs of Untreated Conditions is Limited. U.S. Government Accountability Office. https://www.gao.gov/products/gao-19-274. Accessed June 14, 2022.
47. Ivanova JI, Birnbaum HG, Chen L, et al. Cost of post-traumatic stress disorder vs major depressive disorder among patients covered by medicaid or private insurance. *Am J Manag Care*. 2011;17(8):e314-23.

© 2023 Taylor HL et al. *JAMA Health Forum.*

48. Greenberg PE, Fournier AA, Sisitsky T, Pike CT, Kessler RC. The economic burden of adults with major depressive disorder in the United States (2005 and 2010). *J Clin Psychiatry*. 2015;76(2):155-162.

© 2023 Taylor HL et al. *JAMA Health Forum.*
