# Supplementary material for: Economic Burden Associated With Untreated Mental Illness in Indiana
Source: JAMA Health Forum. 2023 Oct 13;4(10):e233535. doi: 10.1001/jamahealthforum.2023.3535 (PMC10576212; doi:10.1001/jamahealthforum.2023.3535)
Supplement: Supplement 2. — Data Sharing Statement [file jamahealthforum-e233535-s002.pdf]

Data Sharing Statement

Taylor. Economic Burden Associated With Untreated Mental Illness in Indiana. *JAMA Health Forum*. Published October 13, 2023. doi:10.1001/jamahealthforum.2023.3535

Data
Data available: No

Additional Information
Explanation for why data not available: Part of the data is available to access via a web link. However, the Medicaid claims and enrollment data used to analyze direct healthcare expenditures will not be made available.
